# The millennium development goals and household energy requirements in Nigeria

**DOI:** 10.1186/2193-1801-2-529

**Published:** 2013-10-17

**Authors:** Francis I Ibitoye

**Affiliations:** Centre for Energy Research and Development, Obafemi Awolowo University, Ile-Ife, Nigeria

**Keywords:** Millennium development goals, Household energy requirement, LEAP model, Scenario analysis, Non-biogenic household carbon dioxide emissions

## Abstract

Access to clean and affordable energy is critical for the realization of the United Nations’ Millennium Development Goals, or MDGs. In many developing countries, a large proportion of household energy requirements is met by use of non-commercial fuels such as wood, animal dung, crop residues, etc., and the associated health and environmental hazards of these are well documented. In this work, a scenario analysis of energy requirements in Nigeria’s households is carried out to compare estimates between 2005 and 2020 under a reference scenario, with estimates under the assumption that Nigeria will meet the millennium goals. Requirements for energy under the MDG scenario are measured by the impacts on energy use, of a reduction by half, in 2015, (a) the number of household without access to electricity for basic services, (b) the number of households without access to modern energy carriers for cooking, and (c) the number of families living in one-room households in Nigeria’s overcrowded urban slums. For these to be achieved, household electricity consumption would increase by about 41% over the study period, while the use of modern fuels would more than double. This migration to the use of modern fuels for cooking results in a reduction in the overall fuelwood consumption, from 5 GJ/capita in 2005, to 2.9 GJ/capita in 2015.

**Electronic supplementary material:**

The online version of this article (doi:10.1186/2193-1801-2-529) contains supplementary material, which is available to authorized users.

## Introduction

Basic energy requirements in Nigerian households are for lighting, cooking, and energy for household appliances. The form and mix of the requirements may depend on various factors including access, availability and affordability of the energy carriers.

Access to, and availability of conventional energy sources have always been a major discussion in Nigeria (Ibitoye and Adenikinju 2007; Oseni 2012; Oyedepo 2012). Access to electricity for basic services, for example, according to Fulkerson et al. (2005), “can help alleviate poverty, improve health, reduce drudgery, and increase literacy …”, all being vital elements of the millennium development goals (United Nations 2010). Estimates of the International Energy Agency (2009) suggest that about 1.5 billion people worldwide have no access to regular electricity, most of these in developing countries. Using the 1999 and 2006 population census figures published by the National Bureau of Statistics (2009) as basis, the estimated population of Nigeria in 2005 was 137 million people, out of which only 46.72% of households had access to public electricity. This figure, however, does not include the large number of households designated as having access but really do not have electricity for most of the time due to incessant power outages, and must rely on alternative sources for electricity, especially self-generation.

Kerosene and liquefied petroleum gas, LPG, are required for cooking in the households. The price differential of these products makes kerosene the popular choice in many households, and for the fact that kerosene is also more freely available in urban areas and some rural areas. When there is scarcity of kerosene, which happens frequently, the shift is usually towards solid biomass for cooking, especially charcoal and wood ESMAP, The World Bank Group (2005). The high price of LPG and its scarcity makes it unattractive for household cooking. Kerosene is also the fuel of choice for lighting in non-electrified households, and also in many electrified households during power outages.

Wood is freely available in most rural areas of southern Nigeria but the stock declines as one moves up north. For this reason wood is either available at zero cost in some cases, or where there is a price tag, at a relatively low cost compared with other cooking fuels. Wood is undoubtedly a major source of fuel for cooking in many Nigerian homes, both rural and urban. Estimates of the National Bureau of Statistics (2005) suggest that, on the average, about 72% of Nigerian households depend on wood and other non-commercial fuels as the main fuel for cooking. However, according to the World Health Organisation (2012), more than 1.5 million people die every year in ten developing countries, including Nigeria, as a result of indoor air pollution. Despite the well established health and environmental effects caused by continued use of wood for cooking, the desire of the national energy policy (Energy Commission of Nigeria 2003) to promote transition from wood fuel to environmentally benign fuels for cooking may remain impracticable, largely due to poverty and weak policies.

Household energy demand is known to be mostly guided by prices of fuels and appliances, disposable income of households, availability of fuels and appliances, and cultural preferences (Dzioubinsky and Chipman 1999; Zhang and Guo 2013). In Nigerian households, one other factor that influences energy consumption, electricity demand for lighting in particular, is the type of dwelling. In this presentation, a household is used to refer to the group of people or family occupying the same dwelling. The dwelling may consist of just one room, hereafter referred to as one-room household, or several rooms, as in apartments or flats, duplex buildings, or bungalows, similarly referred to as many-room household.

In typical urban areas of Nigeria, a majority of low-income households live in just one-room, and there could be multiple of such households in the same building, with rooms facing a common hallway. Families in such buildings share common utilities such as kitchen and sanitary facilities, and population in each room could at times be in the excess of four people in large urban centres. Because of lack of sufficient living area per person, this type of occupancy may well qualify to be classified as slum dwelling according to the definitions of UN-Habitat (2012) and the United Nations (2012), and it is definitely a case of overcrowding. The one-room dwelling-type could well serve as a proxy for low-income urban dwellers. According to a national poverty profile survey of the National Bureau of Statistics (2005), these one-room households constituted about two-thirds of total number of households in 2004. Electricity requirement for lighting for this dwelling type is certainly lower than for families in bungalows, apartments etc. Overcrowding has its own attendant problems, amongst which are health hazards, increased crime rates, lack of access to modern energy services, lack of access to education etc., all of these being impediments to the achievement of the millennium goals.

This paper focuses on direct energy consumption in Nigerian households, and does not include energy use for transportation, self-generation of electricity, and indirect energy consumptions such as energy for manufacturing and transportation of consumed goods. Furthermore, this paper does not study the effects of energy pricing, and makes no assumption on the ability of the consumer to pay for the energy services.

## Methodology

A scenario-based energy-environment accounting modelling tool, the Long-range Energy Alternatives Planning system, LEAP, developed by the Boston Centre of the Stockholm Environmental Institute (Heaps 2010), has been used to model scenario developments in the Nigerian household sector under various assumptions, with 2005 as the base year. The LEAP model is able to work its way up from energy resource base through extractions, processing, conversion, transmission, up to end-use consumption by demand devices, and under a range of scenario assumptions. Some input data for the LEAP model were processed exogenously using a spreadsheet. The level of disaggregation of demand for energy adopted in the LEAP model is shown in Table 1. The Nigerian household is disaggregated into two broad sectors - urban and rural households. This becomes relevant in view of the urban–rural dichotomy, which is a common feature in many developing countries. Socio-economic conditions in these regions differ to a great 7extent. While the rural population is largely engaged in agricultural activities, the urban population is mostly employed in industrial and services sectors. Access, availability and sometimes pricing of energy carriers also differ between urban and rural areas. The demand categories in Table 1 are the main energy services required in the household while the sub-categories are used to distinguish requirements in dwelling types, when this is likely to have an impact on demand.

**Table 1:** **Disaggregation of the demand for energy in LEAP**

Sector	Sub-sector	Demand category	Demand sub-category
Urban	Electrified HH^*^	Cooking	
		Lighting	One-room HH
			Many-room HH
		Other Electric Appliances	One-room HH
			Many-room HH
	Non-electrified HH	Cooking	
		Lighting	One-room HH
			Many-room HH
Rural	Electrified HH	Cooking	
		Lighting	One-room HH
			Many-room HH
		Other Electric Appliances	One-room HH
			Many-room HH
	Non-electrified HH	Cooking	
		Lighting	One-room HH
			Many-room HH

^*^HH - Household.

For each demand category, LEAP calculates energy demand as the product of an activity level (e.g. number of households) and an energy intensity (or energy use per unit of activity). Energy demand for a demand category in LEAP can be carried out by one of two methods. One is by final energy demand analysis, by way of specifying the activity level and the amount of fuel used per household. The other method is useful energy demand analysis, in which one specifies the useful energy intensity and the efficiencies of end-use devices. The particular route chosen depends on the amount of data available to the analyst.

Two scenario developments are investigated in this study; these are the reference scenario and the MDG scenario.

### Reference scenario, and scenario assumptions

The reference scenario follows the concept of the *most-likely-development-path*, which takes into consideration national policies in terms of future projections and estimates, but does not necessarily project into the future inefficiencies in the current system. Salient features of the reference scenario are highlighted below.

#### Assumptions on population, population projection and dwelling characteristics

Population, population growth rate, and dwelling characteristics are important drivers of energy demand in Nigerian households. Population estimate for 2005 stood at 137 million people, of which 47% reside in urban areas, while the remaining 53% live in rural communities. In this study, future population estimates are based on the medium variant of the 2008 revision of world population as published by United Nations’ Population Division (2010). According to the National Bureau of Statistics (2005), 65.86% of households in Nigeria live in single rooms, while the remaining live in apartments, duplex buildings, bungalows etc., with two or more rooms.

#### Assumptions on energy services

Main energy requirements are for cooking, lighting and non-substitutable electricity (i.e. energy use for electrical appliances such as television sets, refrigerators, air conditioners, etc.).

##### Fuel for cooking

As indicated in section 1, the main fuels for cooking in Nigerian households are kerosene, LPG, charcoal, wood, and electricity, when and where available. Animal and crop residues are also used in some rural communities.

Two sources of data are used to estimate the “energy into the pot” or useful energy for cooking in the household sector. These are the northern Nigerian household survey conducted in 1990 by Silviconsult (1991) covering about 2000 households, and survey conducted by Triple “E” (1992) for some 57 households in Lagos metropolis. While the smaller Lagos survey is representative of a typical metropolis in Nigeria, the Silviconsult survey covered both rural and urban households in northern Nigeria. The two surveys put the useful energy requirement for cooking between 700 and 900 MJ/capita per year, which is close to the estimate for India (WEC/FAO 1999). An average value of 800 MJ/capita per year is assumed in this study.

Energy requirement for cooking is estimated by specifying the useful energy as assumed above, and the efficiencies of cooking devices. In this work, assumed efficiencies for cooking devices are 27.5% for kerosene wick stove, 65% for electric stove, 50% for LPG stove, 17.5% for charcoal stove, and 14% for wood and other biomass stoves. The shares of various energy carriers for cooking may vary from year to year, depending on access, availability and pricing of the energy carriers. Fuel shares used in this work for the base year are based on the need to account for fuels credited to have been consumed in the household sector in the energy balance table of 2005. The fuel shares and data matching were also repeated for subsequent years for which data were available, up till 2008. For 2009 and beyond, the fuel shares adopted for cooking are as published by the National Bureau of Statistics (2005) and these are 69.98% wood, 26.55% kerosene, 1.11% LPG, 0.52% electricity, 0.84% charcoal, and 1% other biomass (i.e. crop residues, animal wastes, etc.). It should be noted that electric cookers are only available in electrified households, while LPG cookers are mainly in urban areas and some rural areas close enough to major cities due to issues of availability of LPG. Future useful energy requirements for cooking are based on the projected population growth, fuel shares and the assumed useful energy intensity.

##### Electricity for lighting and household appliances

The energy carriers most often used for lighting in the households are electricity and kerosene. The contributions of the use of vegetable-oil, candle wax, etc. are assumed negligible. In 2005, 71% of urban dwellers had access to electricity, while only 25% in rural areas had access. On the overall, the national average of access to public electricity was 47% in 2005. The reference scenario assumes that access to electricity will increase nationwide to 60% in 2015, and 65% in 2020; this is in line with the projection of the Energy Commission of Nigeria (Sambo, cited UNDP 2009). It is further assumed in this work that electricity availability factor, EAF (i.e. the fraction of the day for which electricity is available) will improve from the average of 0.64 in 2005 to 1.0 by 2020 in urban areas. In rural areas and for households on the edge of cities or urban slums, EAF is expected to rise from 0.38 in 2005 to 1.0 in 2030. These assumptions are based on current efforts of the national electricity utility to expand generation capacity, and also rehabilitate the national grid and electricity distribution infrastructures.

Electricity requirements for lighting in households is disaggregated into two demand categories based on dwelling types, as indicated in Table 1, that is, one-room households and many-room households. Electricity consumption in these demand categories is estimated based on the type of lighting devices used, the number in use, the number of hours per day when they are used, and the average consumption of the devices per unit time.

Non-substitutable electricity is required for household electrical appliances such as television, refrigerator, air conditioner etc. Since no data is available for this demand category, it is assumed that electricity deemed to have been consumed in the household sector in the energy balance table but not used for cooking or lighting is consumed to run household electrical appliances. This is projected into the future using the number of electrified households, electricity availability factor, and energy intensities of household appliances as the driving parameters.

Electricity requirement for household appliances is also disaggregated into two categories based on dwelling type. This is because of possible duplication of some appliances in many-room households; a many-room household may, for example, have more than one cooling fan, television or air conditioner, and energy requirements for electric appliances would be different for the two dwelling types. Estimates of energy intensities are based on average number of electric appliances in each household, average number of hours of use and average energy consumption. On this basis, the energy intensities for basic household appliances are 285 and 395 kWh/annum respectively for one-room and many-room households. Basic appliances here include pressing iron, radio, television and cooling fan. For refrigeration and air conditioning, the estimated energy intensities are 402 and 4600 kWh/annum, and respectively. According to a survey of the National Bureau of Statistics (2005), however, only 20% of households had at least one air conditioner and/or a refrigerator.

##### Kerosene for lighting

Kerosene is the sole fuel for lighting in non-electrified households; it is also the fuel of choice in electrified households during the periods of power outages. Future consumption of kerosene for lighting in both electrified and non-electrified households in urban and rural dwellings is tied to the number of households in these demand categories, electrification rates, and electricity availability factors as defined earlier.

Estimate of kerosene consumption for lighting is based on an energy intensity of 1.22 Gigajoule/lantern/year, the average number of households in each demand category, and the average number of lanterns per dwelling type.

### The MDG scenario, and scenario assumptions

Three separate development paths in the residential sector are employed to build up the millennium development goals (MDG) scenario, and the various ways by which these development paths contribute to the achievement of the MDGs have been documented (United Nations 2005; UNDP [Bibr CR14][Bibr CR15][Bibr CR16]). The development paths investigated under the MDG scenario are:

i. *Improved electricity access*, whereby it is desired to reduce by half, in 2015, the number of households without access to electricity for basic services. The impact will be mostly felt in rural areas, since as at 2005, only 25% of rural dwellers had access to electricity and 71% in urban areas. This means that by 2015, the number of rural dwellers without access will be about 38%, and 14% in urban households.
ii. *Improved living condition in urban areas*, by way of reducing overcrowding or the number of one-room households in urban areas. This has direct bearing on electricity consumption, since the demand for electricity for lighting and electrical appliances is more for a multi-room dwelling type. The assumption here is that we reduce by half, in 2015, the number of families in one-room urban households. This assumption is not extended to rural communities since these are usually characterized by low population densities.
iii. *Access to clean modern fuels for cooking*, treated by assuming that electrified households in rural or urban areas have reached some level of social development that enables them to have easy access to modern fuels but still rely on solid fuels to some extent for economic or other reasons. The term *modern fuels* as used here is as defined by UNDP (2009), and refers to electricity, liquid fuels (such as kerosene), and gaseous fuels such as LPG or natural gas), but excludes traditional biomass and coal. In contrast, non-electrified households are largely in rural areas with no access to electricity and of lower social development, and most likely, no access to modern cooking fuel such as LPG or kerosene because of their high price tags and perennial scarcities. Thus the desire to improve access to modern fuels may not be popular in non-electrified rural areas, where access to cheap or zero-cost solid fuels is unrestricted, and in a case where the use of kerosene for cooking and lighting still needs to be promoted by policy measures to reduce dependence on solid fuels, especially wood. Hence we assume, in this development path that we reduce by half in 2015 the number of households without easy access to modern cooking fuels in all electrified households, both urban and rural. As earlier indicated, this paper makes no assumptions about the ability of this category of consumers to pay for modern energy services.

## Results and discussion

### Improved electricity access (IEA)

**Figure 1:**
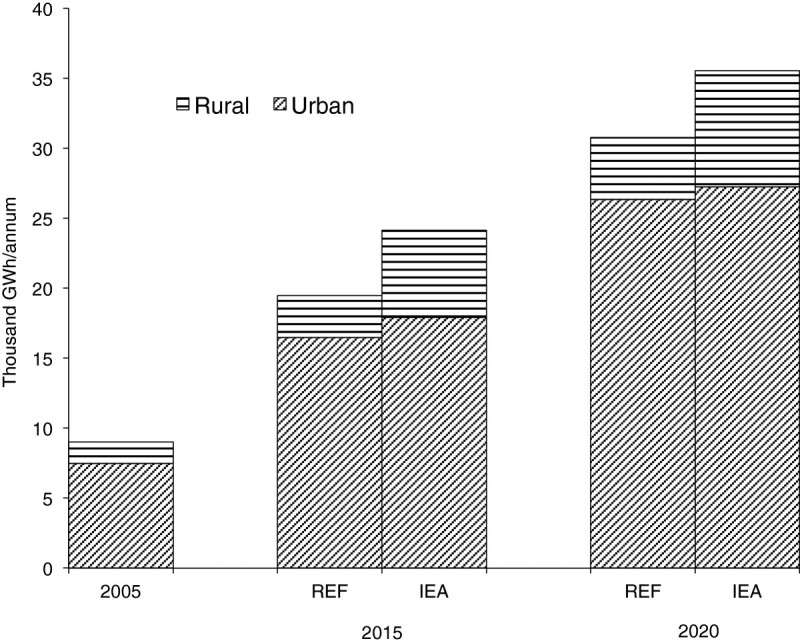
**Demand for electricity in reference (REF) and improved electricity access (IEA).**

**Figure 2:**
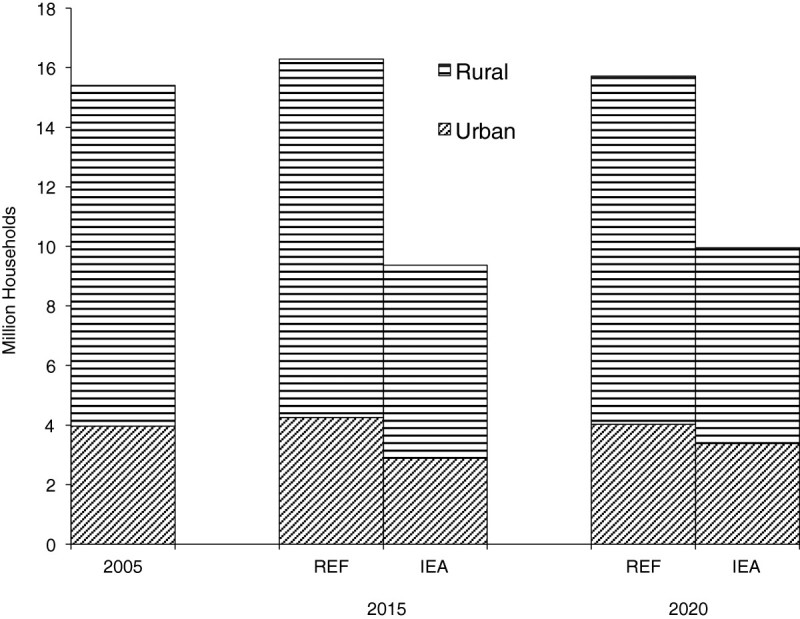
**Number of households with no access to electricity in rural and urban areas in reference (REF) and improved electricity access (IEA).**

Electricity requirements under the improved electricity access option are compared with the reference scenario (REF) in Figure 1. Over the study period, the demand for electricity is expected to increase by roughly 17% over the reference case, a majority of the increase resulting from electricity for basic needs in rural areas. While access increased marginally in urban areas, it more than doubles in rural areas in 2015 and 2020. We note that the 17% increase observed here, though not spectacular, is actually the increase beyond the national target in the reference scenario. The number of households without access to electricity for each of the scenarios is shown in Figure 2. We observe that the number of households without electricity reduces by half in 2015 as postulated.

### Improved living condition (ILC)

**Figure 3:**
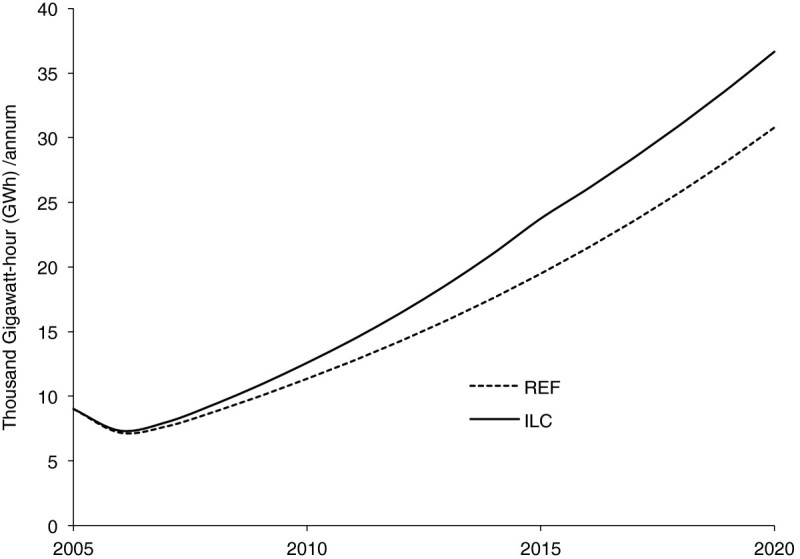
**Electricity requirements for reduction of overcrowding in urban areas under reference (REF) and improved living condition (ILC).**

Reduction of overcrowding in urban areas by half in 2015 is assumed to translate to a reduction of the number of families living in one-room households by half in the same year. As already discussed, the main effect on energy needs will be on the demand for electricity, and this is shown in Figure 3. Over the study period the demand for electricity will increase by nearly 16%, mainly for lighting and non-substitutable electricity for household electrical appliances.

### Improved access to modern fuels for cooking, (MFC)

**Figure 4:**
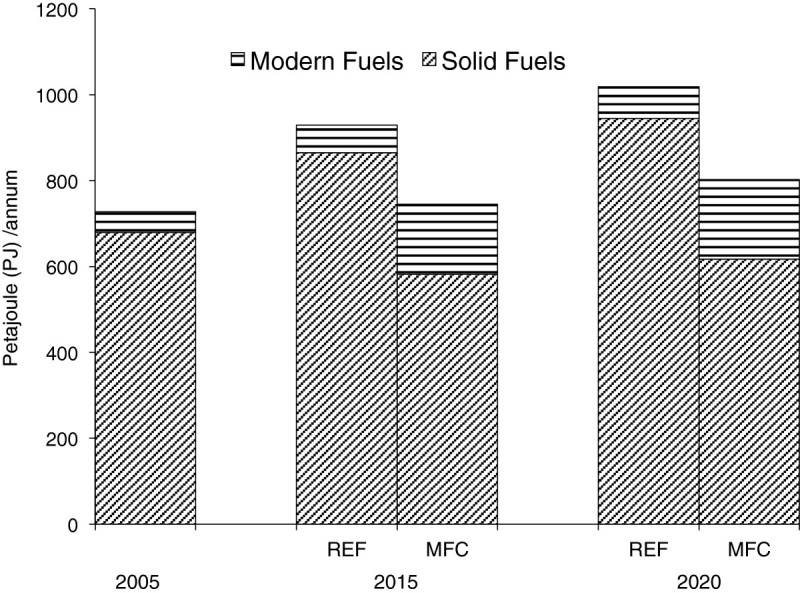
**Final energy requirements for solid and modern fuels for cooking in reference (REF) and improved access to modern fuels for cooking (MFC).**

Final energy requirement for cooking with improved access to modern fuel is shown in Figure 4. As earlier mentioned, increase in the use of modern fuels can only be meaningful in urban areas where access and availability to modern fuels can be assured to some extent, and in some rural areas, especially if these are electrified and close to urban centres. The reference scenario assumes a gradual decline in solid fuel dependence while the MFC option ensures a steady climb along the energy ladder towards more efficient fuels. As expected, Figure 4 shows a displacement of solid fuels by modern fuels.

### The millennium development goals (MDG) scenario

**Figure 5:**
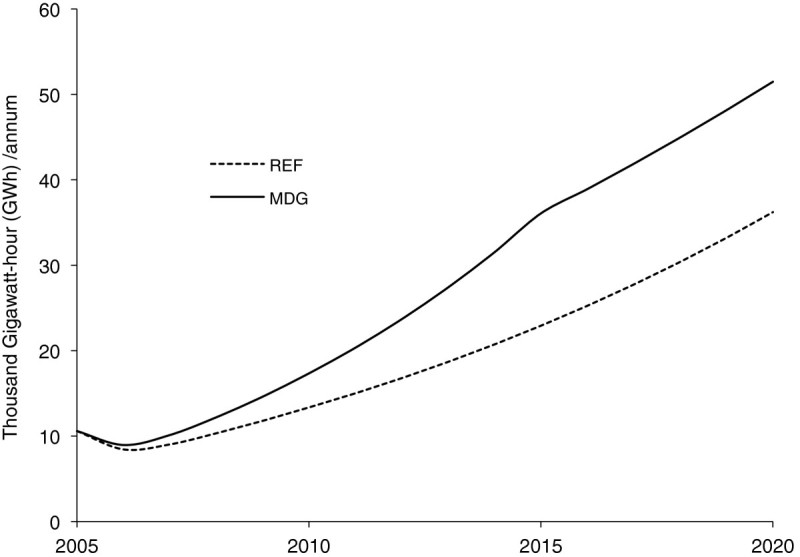
**Electricity generation under reference (REF) and millennium development goals (MDG) scenarios.**

**Figure 6:**
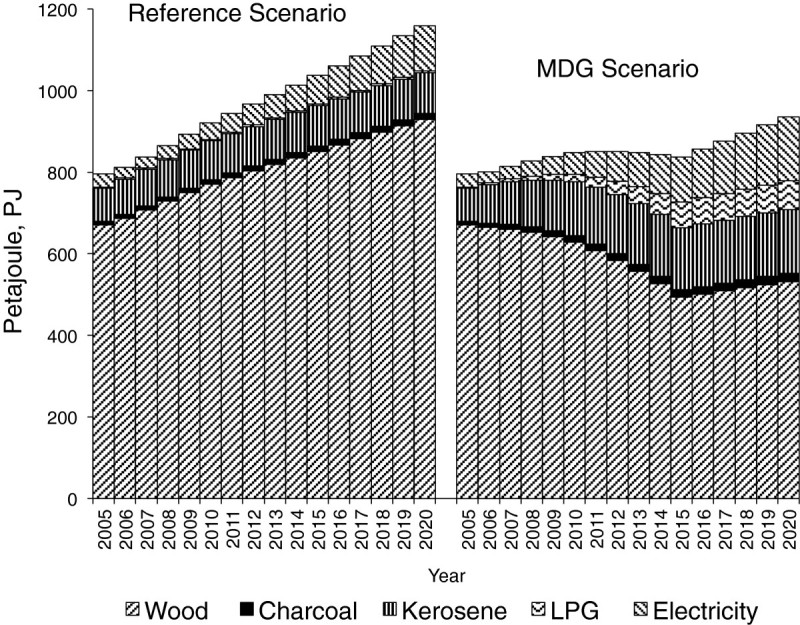
**Household final energy requirement under reference and millennium development goals (MDG) scenarios.**

**Figure 7:**
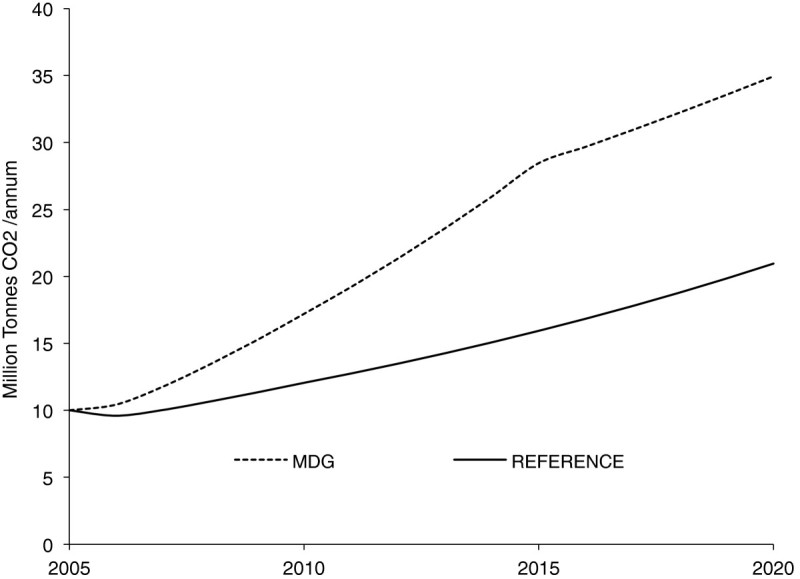
**Impact of energy use on non-biogenic carbon dioxide emissions in reference and millennium development goals (MDG) scenarios.**

The MDG scenario is the combined effects of the three development options discussed above, all implemented simultaneously, that is, improved access to electricity, improved living conditions, and improved access to modern fuels. The impacts on energy use are shown in Figures 5,6,7, in terms of the demand for electricity, final energy requirements, and carbon dioxide (CO_2_) emissions emanating from the household sector under the reference and MDG scenarios. In Figure 5 cumulative electricity requirements over the study horizon increases by about 41% under the MDG scenario. The aggregate per capita household electricity consumption under the MDG scenario would increase from 66 kWh/cap in 2005 to 174 kWh/cap in 2015, compared with the 111 kWh/cap under the reference scenario, same year.

The household energy-mix under the two scenarios as shown in Figure 6 indicates a gradual displacement of solid fuels by cleaner fuels. Under the MDG scenario, while there are increases over the reference case in the consumption of electricity, kerosene and LPG, we notice a steady decline in the consumption of traditional biomass. Fuelwood consumption per capita reduces from 5 GJ/cap in 2005 to 2.9 GJ/cap in 2015. This becomes relevant in view of the fact that wood consumption is an important indicator for environmental sustainability under the millennium development objectives. The observed reduction in total final energy requirement under the MDG scenario over the study period in Figure 6 is the result of a transition to more energy-efficient fuels for cooking.

The impact of energy use on non-biogenic household CO_2_ emissions under the reference and MDG scenarios is illustrated in Figure 7. On the overall, there is a 56% increase in cumulative non-biogenic household CO_2_ emissions under the MDG scenario over the entire study period. About 37% of this increase is due to the combustion of natural gas for electricity production, while a large proportion of the remaining emissions can be attributed to those arising from the transition from wood (of zero net emissions) to modern fuels for cooking.

One pertinent question arising is that, inspite of the environmental burden of a 56% increase in CO_2_ emissions, is the pursuit of the MDGs still worthwhile? In this analysis, all additional future requirements for electricity generation are met using natural gas plants. The use of natural gas plants for power production appears to be the *most-likely-development* pathway in view of the fact that Nigeria has a large reserve of natural gas, the eighth largest reserve in the world. However, Nigeria formally embraced low-carbon development strategy in 2011, which means that the country will have to start seeing its abundant renewable energy resources, especially hydro (about 15 GWe) and solar energy (3.5 -7.0 kWh/m^2^-day) as major players in an effort to meet its increasing needs for electricity production. The increased CO_2_ emissions from improving access could well be compensated by reduced emissions through energy efficiency and increased use of renewable energy in the residential and other sectors (Volpi et al. 2006). If this happens, then the environmental burden of CO_2_ emissions in the pursuit of MDGs will be greatly reduced. Also, according to Figure 7, household CO_2_ emissions grew from 9.99 million tonnes (73 kg per capita) in 2005 to 34.94 million tonnes (i.e. 181 kg/capita) by 2020 under the MDG scenario. When compared with national and global emissions reported in human development report (UNDP 2007), it can be safely said that the task of meeting energy needs for millennium goals in Nigerian households only contribute a very small proportion to national or indeed global emissions.

## Conclusions

A scenario analysis for the demand for energy in Nigerian households in support of United Nations’ millennium development goals have been carried out using three development paths - improved access to electricity, improved access to modern fuels for cooking, and improved living conditions for the overcrowded urban slums. Analyses show that, in order to achieve the millennium goals, demand for electricity in the households will increase by 41% over the reference scenario, while the demand for modern fuels for cooking will more than double, leading to a reduction in fuelwood consumption. However, one direct consequence of these development options is a 56% increase in cumulative non-biogenic CO_2_ emissions. A combination of household energy efficiency improvement programme and the adoption of low-carbon energy technologies, especially renewable energy options on the energy supply side will ensure that the benefits of meeting the energy needs of the millennium goals outweigh the environmental penalties.

## Authors’ original submitted files for images

Below are the links to the authors’ original submitted files for images. Authors’ original file for figure 1 Authors’ original file for figure 2 Authors’ original file for figure 3 Authors’ original file for figure 4 Authors’ original file for figure 5 Authors’ original file for figure 6 Authors’ original file for figure 7

## Abbreviations

CO2: Carbon dioxide
EAF: Electricity availability factor
FAO: Food and agricultural organisation
GDP: Gross domestic product
GJ: Gigajoules
IEA: Improved electricity access
ILC: Improved living condition
LEAP: Long-range energy alternatives planning system
LPG: Liquefied petroleum gas
MDG: Millennium development goals
MFC: Modern fuels for cooking
PJ: Petajoules
UNDP: United Nations development programme
WEC: World energy council.
