# Supplementary figures and images for: The millennium development goals and household energy requirements in Nigeria
Source: Springerplus. 2013 Oct 17;2(1):529. doi: 10.1186/2193-1801-2-529 (PMC3825065; doi:10.1186/2193-1801-2-529)

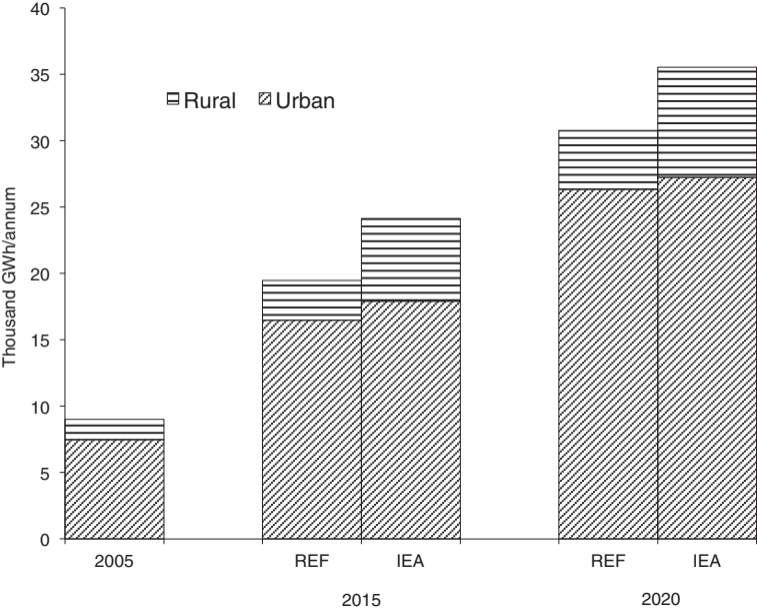

Supplement: Supplementary file 1 — Authors’ original file for figure 1 [file 40064_2013_601_MOESM1_ESM.pdf]

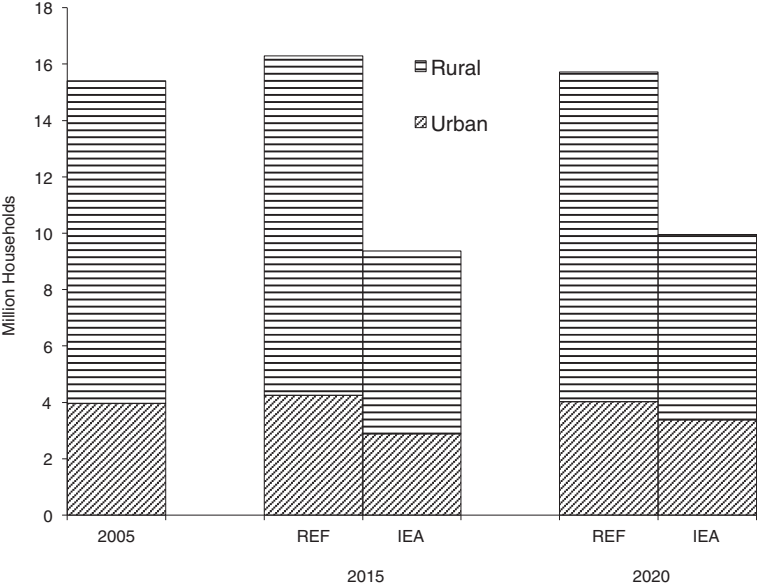

Supplement: Supplementary file 2 — Authors’ original file for figure 2 [file 40064_2013_601_MOESM2_ESM.pdf]

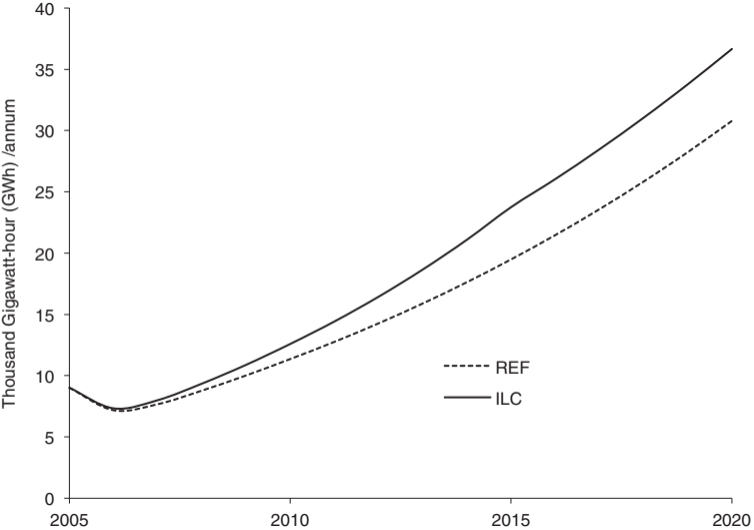

Supplement: Supplementary file 3 — Authors’ original file for figure 3 [file 40064_2013_601_MOESM3_ESM.pdf]

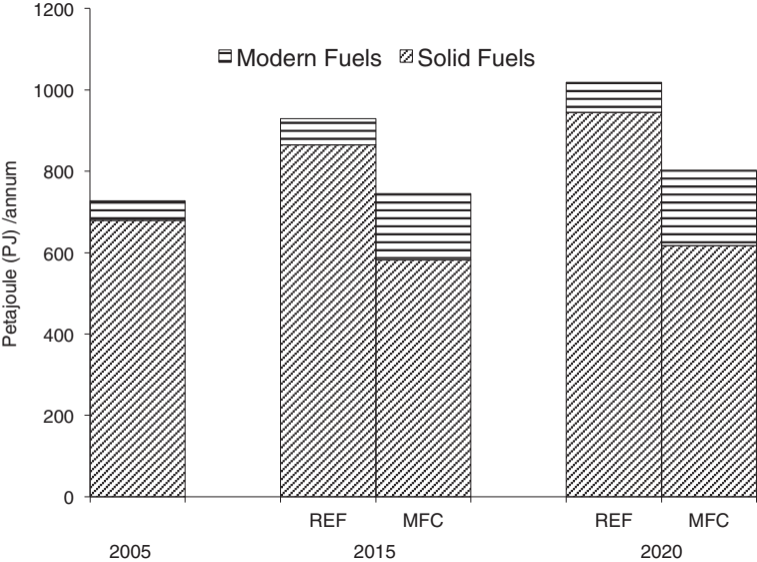

Supplement: Supplementary file 4 — Authors’ original file for figure 4 [file 40064_2013_601_MOESM4_ESM.pdf]

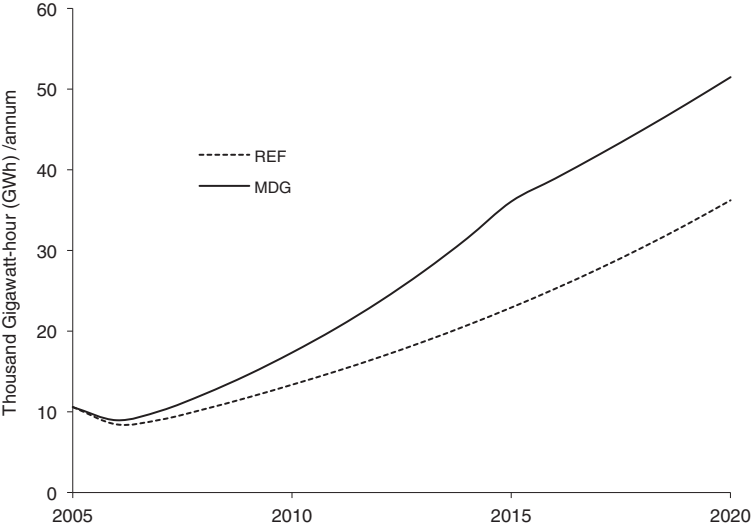

Supplement: Supplementary file 5 — Authors’ original file for figure 5 [file 40064_2013_601_MOESM5_ESM.pdf]

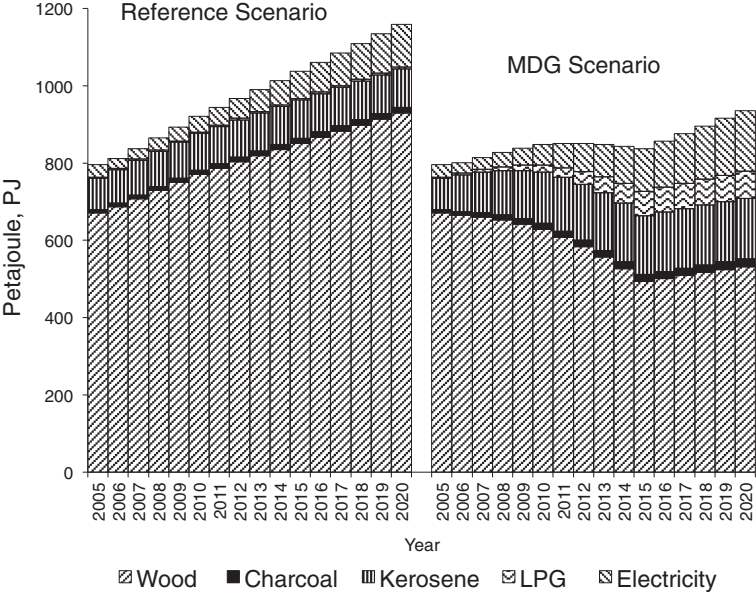

Supplement: Supplementary file 6 — Authors’ original file for figure 6 [file 40064_2013_601_MOESM6_ESM.pdf]

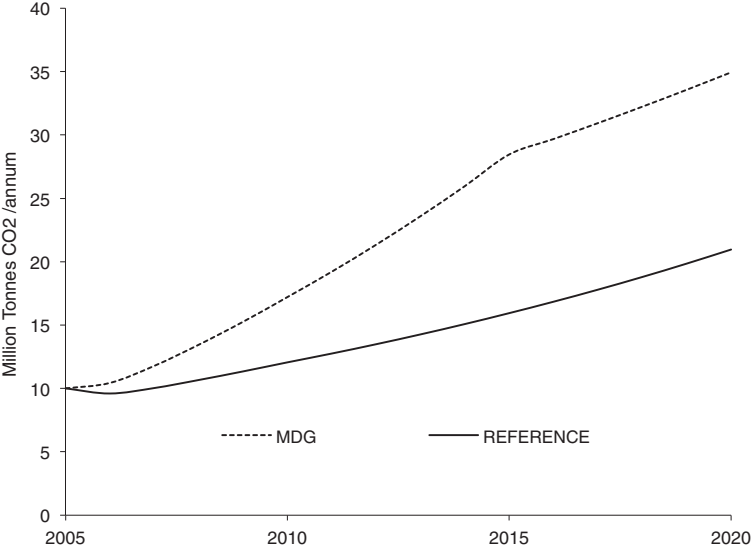

Supplement: Supplementary file 7 — Authors’ original file for figure 7 [file 40064_2013_601_MOESM7_ESM.pdf]
